# *SGK1* mutation status can further stratify patients with germinal center B‐cell‐like diffuse large B‐cell lymphoma into different prognostic subgroups

**DOI:** 10.1002/cam4.4550

**Published:** 2022-02-01

**Authors:** Baoping Guo, Yujie Huang, Ying Duan, Chengcheng Liao, Hong Cen

**Affiliations:** ^1^ Department of Chemotherapy Guangxi Medical University Affiliated Tumor Hospital Nanning China

**Keywords:** classification, diffuse large B‐cell lymphoma, genetics, prognosis, SGK1

## Abstract

There are over a 100 driver gene mutations in patients with diffuse large B‐cell lymphoma (DLBCL), but their clinical significance remains unclear. Here, we first analyzed the DLBCL dataset from the UK‐based Haematological Malignancy Research Network. Patients were divided into high‐ and low‐risk groups based on whether lymphoma progressed within 24 months. Genes showing significantly different frequencies between groups were selected. Survival data for patients with the selected mutant genes were analyzed. The results were validated using two other large databases to evaluate the relationship between the selected mutant genes and prognosis. The mutation frequencies of 11 genes (*MYD88*[L265P], *SGK1*, *MPEG1*, *TP53*, *SPEN*, *NOTCH1*, *ETV6*, *TNFRSF14*, *MGA*, *CIITA*, and *PIM1*) significantly differed between the high‐ and low‐risk groups. The relationships between these mutant genes and patient survival were analyzed. Patients who harbored *SGK1* (serum and glucocorticoid‐inducible kinase 1) mutations exhibited the best prognosis. Most patients with SGK1 mutation are germinal center B‐cell (GCB) subtype. Among patients with GCB DLBCL, those harboring *SGK1* mutations exhibited better prognosis than those without *SGK1* mutations. Most *SGK1* mutations were single‐base substitutions, primarily scattered throughout the catalytic domain‐encoding region. Multiple *SGK1* mutations were identified in a single patient. Thus, *SGK1* mutations are a marker of good prognosis for DLBCL and occur predominantly in the GCB subtype of DLBCL. *SGK1* mutation status can further stratify patients with GCB DLBCL into different prognostic subgroups.

## Graphical abstract

We believe that our study makes a significant contribution to the literature and will be of interest to the readership of your journal, because it provides strong evidence that mutations in serum and glucocorticoid‐inducible kinase 1 (SGK1) are associated with better prognosis in patients with diffuse large B‐cell lymphoma and, thus, that targeting SGK1 may improve patient prognosis.

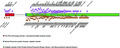

## 1 INTRODUCTION

Diffuse large B‐cell lymphoma (DLBCL) is the most common type of lymphoma. The most frequently used clinical indicators for the prognosis of DLBCL include the International Prognostic Index, which predicts prognosis based on patient clinical characteristics, and indicators that predict prognosis based on the cell of origin (COO) (namely the germinal center B‐cell [GCB] and activated B‐cell [ABC] subtypes of DLBCL). Widespread clinical use of next‐generation sequencing has led to the discovery of more than 100 cancer driver genes associated with lymphoma. Several recent studies have sought to introduce the use of cancer driver gene mutations for the molecular classification of DLBCL.^1^, ^2^, ^3^ However, these studies were retrospective and used complicated classification procedures based on computational algorithms. Additionally, standardized criteria were not used among these studies, and the same gene mutation appeared in different molecular subtypes, rendering the results difficult to apply prospectively for subtype classification in patients. Furthermore, limited information is available on each mutant gene; thus, it is difficult to utilize the mutants accurately to guide clinical treatment. Except for the *MYD88*(L265P) mutation, which appears to be clinically important (patients with this mutation have a poor prognosis),^4^, ^5^ knowledge on other DLBCL gene mutations is limited. Based on extensive sequencing efforts worldwide and identification of common DLBCL mutations, future research should focus on interpreting the available mutation data. To this end, we analyzed the datasets containing detailed clinical and gene mutation data published in high‐impact journals, to identify clinically significant mutant genes that can predict prognosis and treatment effectiveness in DLBCL.

## 2 MATERIALS AND METHODS

### 2.1 DLBCL cohorts

Datasets from three published articles were reanalyzed in this study. The gene mutation and clinical data are provided in Supporting Information. The discovery patient cohort was from the Lacy et al. Dataset (S2),^3^ and the gene mutation data and detailed patient clinical data are included in Supporting Information of their study. Patients in the Lacy et al. dataset were derived from the UK population‐based Haematological Malignancy Research Network (HMRN; https://www.hmrn.org). The validation datasets included cohorts from studies conducted by Reddy et al. (Dataset S3)^6^ and by Chapuy et al. (Dataset S1)^2^

### 2.2 Analysis methods

The discovery dataset was evaluated using data from the HMRN reported by Lacy et al. The HMRN dataset has a large sample size and contains comprehensive clinical data. We first selected patients who were followed up for 24 months or longer; based on their Progression of Disease within 24 Months (POD24), we divided these patients into high‐ and low‐risk groups. Chi‐square test was used to analyze the frequencies of different mutated genes in the groups, and genes showing significant differences in frequency between groups were selected. Survival curves were plotted to identify mutations impacting prognosis. The results were validated using published data by Reddy et al. and Chapuy et al. The relationships between selected gene mutations and DLBCL subtypes were analyzed.

### 2.3 Lollipop plot of mutation sites in *SGK1*

serum and glucocorticoid‐inducible kinase 1 (*SGK1*) mutation data from the DLBCL cohort from Lacy et al. were downloaded. Data analysis was conducted using R version 3.5.2 (R Core Team). After eliminating mutations that did not lead to protein changes, the sites of gene mutations and frequencies of genes containing these mutations were obtained. Lollipop plots were drawn using the R package Maftools^7^ and trackViewer^8^ to illustrate the sites of mutations in *SGK1* and corresponding sites in the SGK1 protein. The sequences of *SGK1* splice isoforms were obtained from the UniProt website (http://www.uniprot.org). We compared these amino acid sequences and found the identification number of the *SGK1* splice isoform corresponding to that reported in the article to be O00141‐2 (RefSeq code NM_001143676). Based on this information, the sequences of the protein domains of the *SGK1* splice isoform were obtained from the UniProt website. Annotations of the protein domains obtained were loaded into a GRanges object, along with the mutation site data.

### 2.4 Statistical analysis

Statistical analyses were performed using GraphPad Prism version 7.00 (GraphPad, Inc.) and R version 3.5.2 (R Core Team) statistical software. Pearson's chi‐square test was employed to compare categorical data. Survival analyses were performed using Kaplan–Meier plots and a log‐rank test. A two‐sided *p* value <0.05 was considered to indicate significant results, unless otherwise stated.

## 3 RESULTS

### 3.1 DLBCL patients with POD24 have a poor prognosis

**FIGURE 1:**
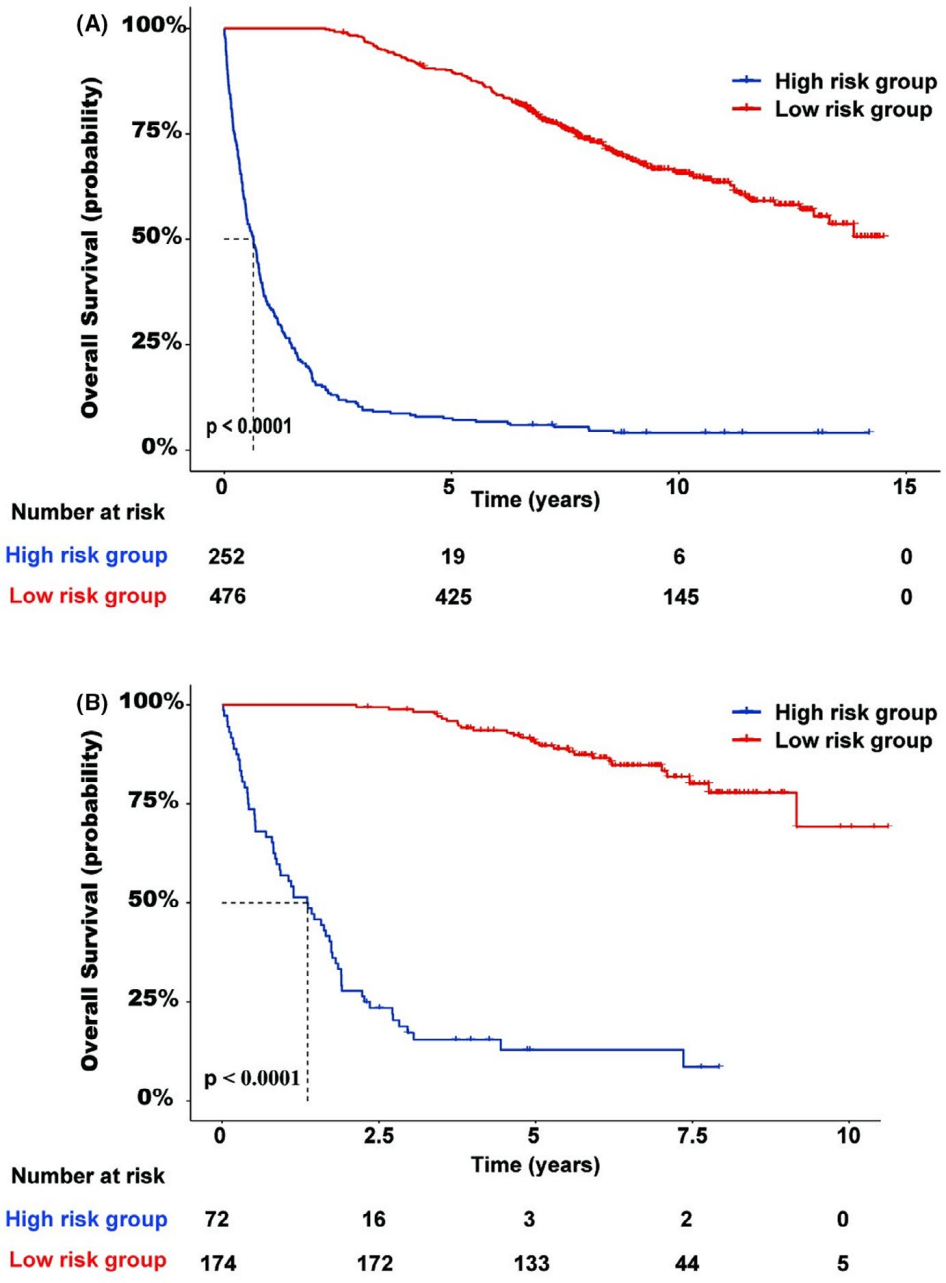
Overall survival of high‐ and low‐risk subgroups identified by Progression of Disease within 24 Months. (A) Cohort from Lacy et al. study and (B) cohort from Chapuy et al. study

Based on our definitions, the Lacy et al. dataset included 252 patients in the high‐risk group and 476 patients in the low‐risk group, whereas the Chapuy et al. dataset included 72 patients in the high‐risk group and 174 patients in the low‐risk group. The survival curves of the two cohorts stratified by risk group are shown in Figure 1. The results showed that patients with POD24 had an inferior overall survival (OS) compared with those without POD24 (*p* < 0.001).

### 3.2 Frequencies of mutated genes in high‐ and low‐risk groups and their relationship with prognosis

The gene mutation data in the Lacy et al. dataset were analyzed, and the mutation frequencies of different genes in the high‐ and low‐risk groups were compared. The mutation frequencies of 11 genes, namely *MYD88*(L265P), *SGK1*, *MPEG1*, *TP53*, *SPEN*, *NOTCH1*, *ETV6*, *TNFRSF14*, *MGA*, *CIITA*, and *PIM1*, significantly differed between groups (Table 1).

**TABLE 1:** Mutant genes with significant differences in frequencies between the high‐ and low‐risk groups (data source: Lacy et al. dataset)

Gene name	Good prognosis (*n*/%)	Poor prognosis (*n*/%)	*p*‐value
*MYD88L265P*	48 (10.08)	50 (19.84)	0.00038
*SGK1*	95 (19.96)	24 (9.52)	0.00044
*MPEG1*	14 (2.94)	19 (7.54)	0.00804
*TP53*	75 (15.76)	60 (23.81)	0.01048
*SPEN*	11 (2.31)	15 (5.95)	0.02095
*NOTCH1*	7 (1.47)	11 (4.37)	0.03221
*ETV6*	23 (4.83)	23 (9.13)	0.03521
*TNFRSF14*	95 (19.96)	34 (13.49)	0.03830
*MGA*	13 (2.73)	1 (0.40)	0.04256
*CIITA*	9 (1.89)	12 (4.76)	0.04893
*PIM1*	112 (23.53%)	77 (30.56%)	0.04904

**FIGURE 2:**
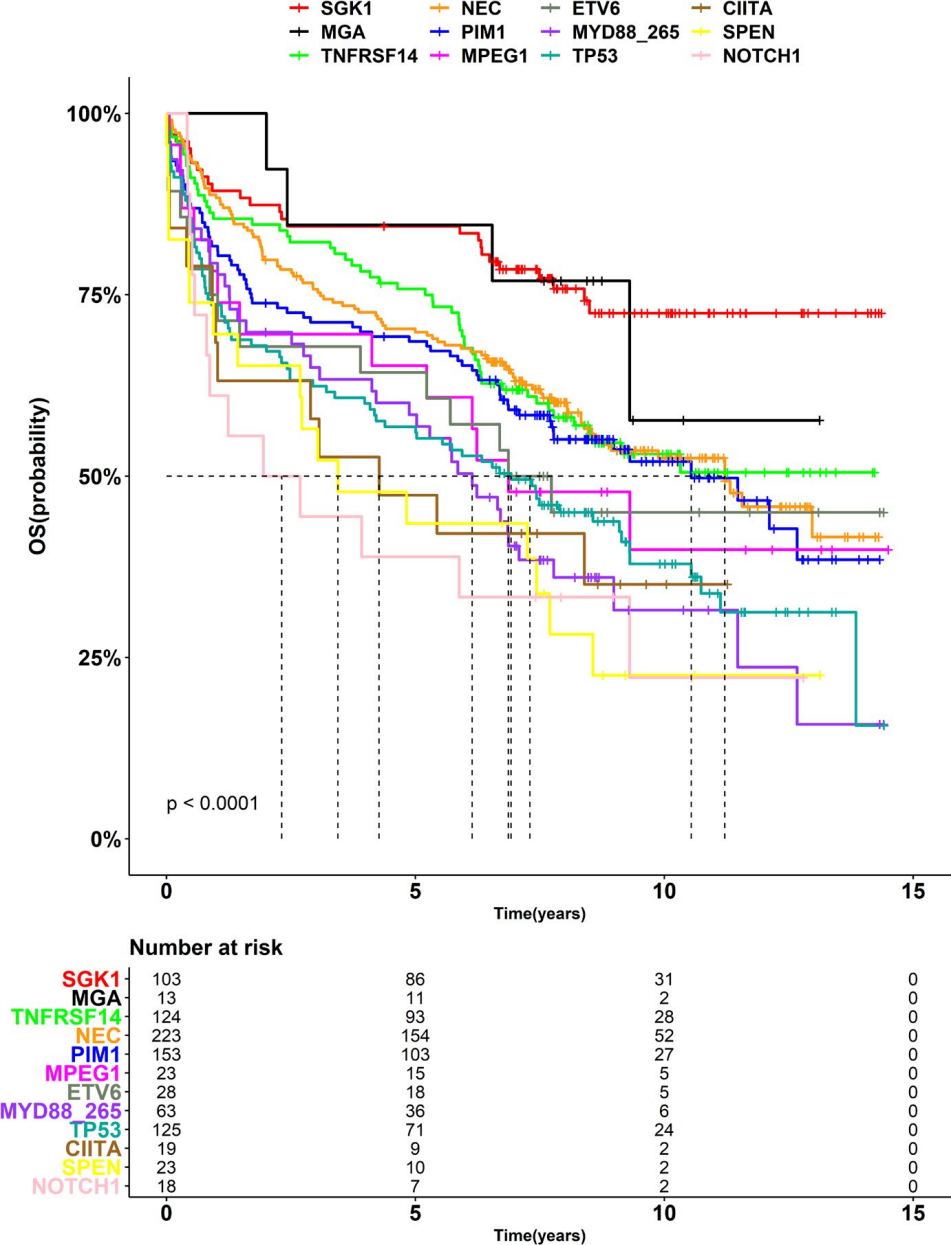
Overall survival of patients harboring different mutant genes (dataset from Lacy et al.)

Next, we investigated the relationships between the selected mutant genes and prognosis by analyzing data from the patients who were administered the R‐CHOP regimen. OS was compared between patients with these 11 mutant genes and those without mutations in any of the abovementioned genes (i.e., not elsewhere classified group). Patients harboring mutations in different genes were assigned to multiple groups. Patients in the *SGK1* mutation group exhibited the best prognosis (*SGK1* was commonly mutated in DLBCL) (Figure 2).

**FIGURE 3:**
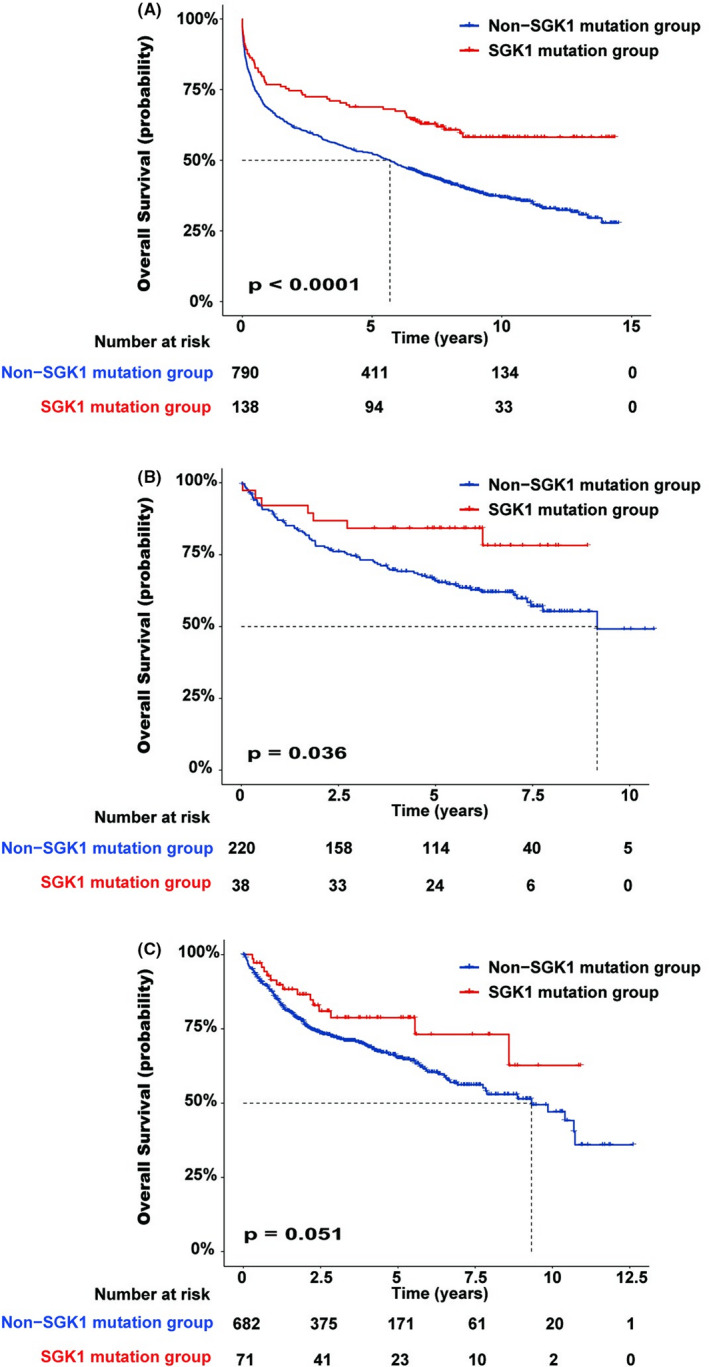
Overall survival of patients with DLBCL with and without *SGK1* mutations. (A) Cohort from Lacy et al. study, (B) cohort from Chapuy et al. study, and (C) cohort from Reddy et al. study

We analyzed two additional datasets to confirm that patients with *SGK1* mutations exhibited a good prognosis (Figure 3).

### 3.3 Relationship between *SGK1* mutations and COO

Data from patients with *SGK1* mutations were extracted from the three datasets and the COO of DLBCL was analyzed. Among the patients whose DLBCL had a clear COO, most had the GCB DLBCL subtype (Table 2).

**TABLE 2:** Relationship between *SGK1* mutations and COO

Study	Number of *SGK1* mutations	GCB	ABC	Unknown	*p*
Lacy et al.	138	66	6	65	<0.0001
Chapuy et al.	38	25	3	10	<0.0001
Reddy et al.	93	51	11	31	<0.0001

Abbreviations: ABC, activated B‐cell; COO, cell of origin; GCB, germinal center B‐cell.

### 3.4 Effects of *SGK1* mutations on the prognosis of GCB and novel DLBCL molecular subtypes

**FIGURE 4:**
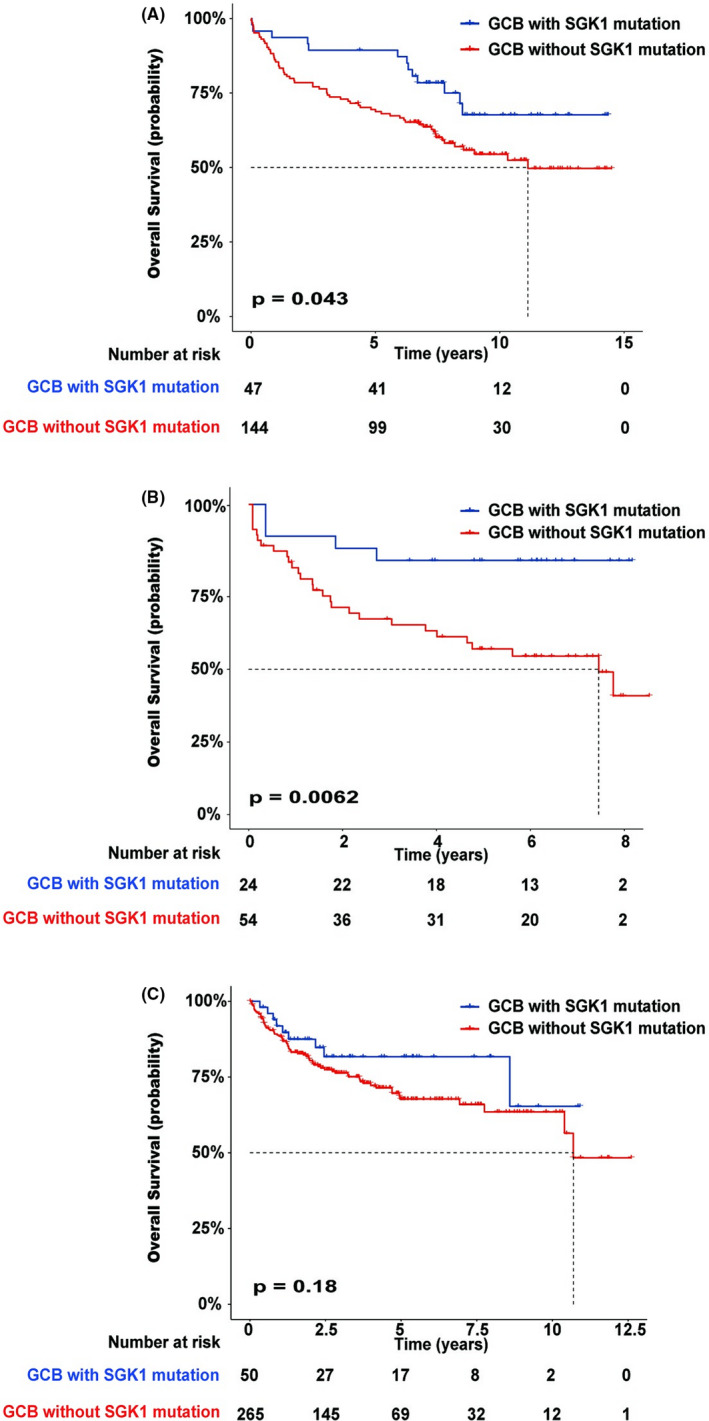
Overall survival of patients with germinal center B‐cell (GCB)‐diffuse large B‐cell lymphoma with and without *SGK1* mutations. (A) Cohort from Lacy et al. study, (B) cohort from Chapuy et al. study, and (C) cohort from the Reddy et al. study

The current standard first‐line treatment for patients with DLBCL is the R‐CHOP regimen. We analyzed the Lacy et al. dataset by selecting 191 patients with the GCB DLBCL subtype who were administered the R‐CHOP regimen. The patients were divided into two groups based on their *SGK1* mutation status, and survival curves were plotted. The results showed that the patients with GCB and *SGK1* mutations exhibited a better prognosis than those without *SGK1* mutations, indicating that GCB patients can be further stratified by their *SGK1* mutation status. Similar results were obtained after the analysis of the Chapuy et al. dataset (Figure 4).

**FIGURE 5:**
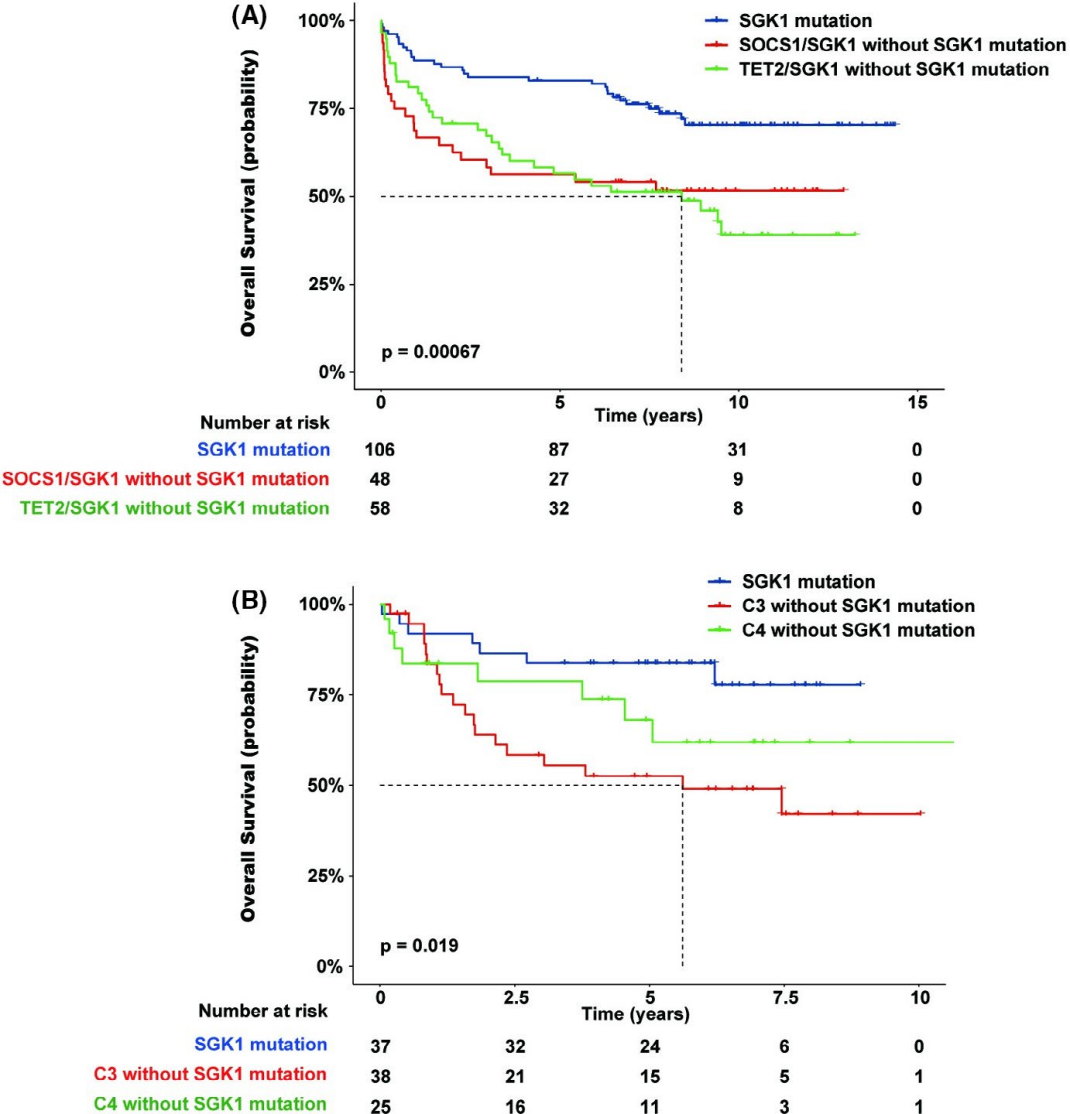
(A) Overall survival of SOCS1/SGK1 and TET2/SGK1 clusters with and without *SGK1* mutations. (B) Overall survival of C3 and C4 subsets with and without *SGK1* mutations

In the Lacy et al. dataset, DLBCL was classified into five clusters using the Akaike Information Criterion, namely MYD88, BCL2, SOCS1/SGK1, TET2/SGK1, and NOTCH2, along with a cluster of unclassified gene mutations. Most patients with *SGK1* mutations were classified into the SOCS1/SGK1 or TET2/SGK1 clusters. The COO in these two clusters was primarily the GCB DLBCL subtype, which was also the molecular subtype associated with good prognosis. We reclassified the patients into three groups: (1) Patients in the SOCS1/SGK1 and TET2/SGK1 clusters with *SGK1* gene mutations; (2) Patients in the SOCS1/SGK1 cluster without *SGK1* mutations; and (3) Patients in the TET2/SGK1 cluster without *SGK1* mutations. After eliminating data from patients in the SOCS1/SGK1 and TET2/SGK1 clusters with *SGK1* mutations, we found that patients in the two clusters without *SGK1* mutations had a poor prognosis (Figure 5A).

The Chapuy et al. dataset used nonnegative matrix factorization consensus clustering for 158 identified driver gene mutations in DLBCL. They discovered five subsets of patients with discrete genetic signatures (C1–C5) and an additional subset of patients without detectable driver gene mutations (C0). The C3 and C4 subsets in the Chapuy et al. dataset were primarily of the GCB DLBCL subtype. We analyzed the C3 and C4 subsets from their study and found that the patients in the C3 and C4 subsets who were administered the R‐CHOP regimen and possessed *SGK1* mutations exhibited a better prognosis than those without *SGK1* mutations (Figure 5B).

### 3.5 Characteristics of *SGK1* mutations

**FIGURE 6:**
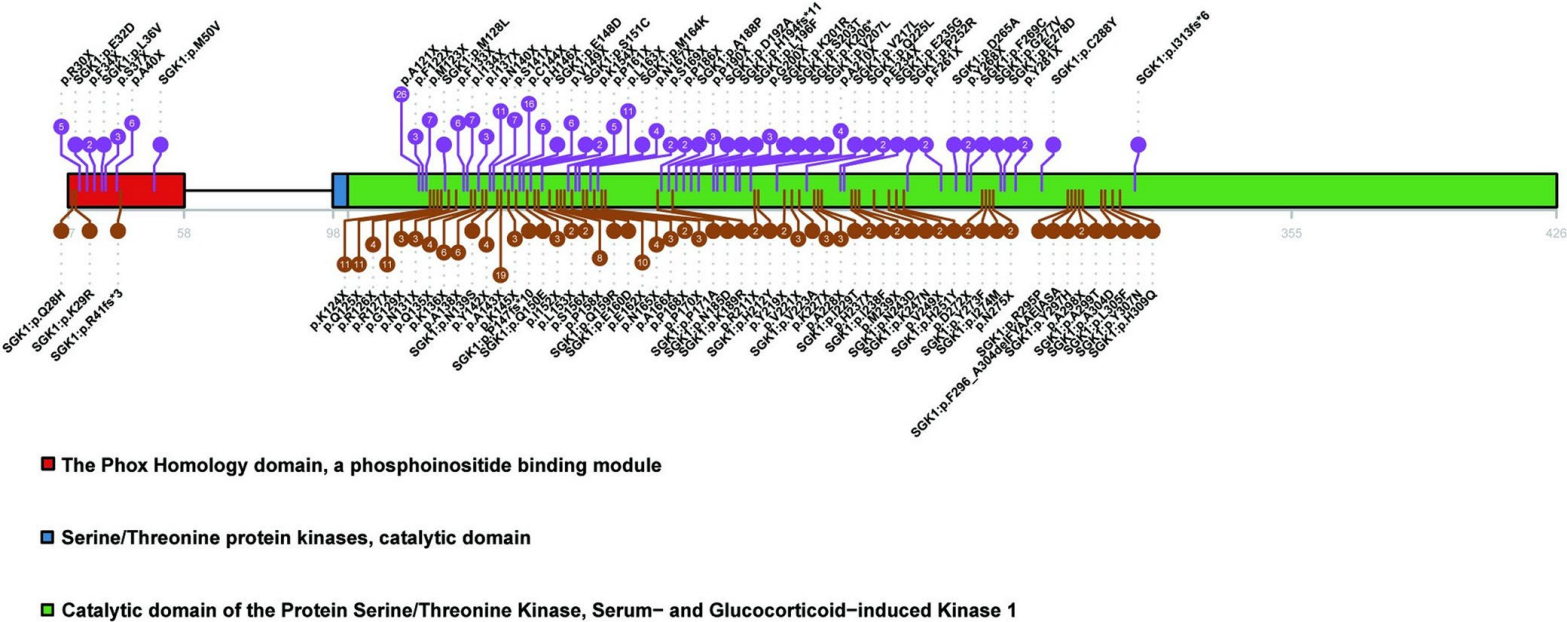
Distribution of *SGK1* mutations. Vertical lines at the top and bottom of the figure indicate the positions of the respective missense mutations causing diffuse large B‐cell lymphoma, and numbers in the circles at the end of vertical lines represent the frequencies of the mutations

Using the Lacy et al. dataset, we identified the mutated proteins encoded by mutant *SGK1*, enabling further analysis of the characteristics of these *SGK1* mutants. Most *SGK1* mutations were single‐base substitutions, with a few being small deletions. *SGK1* mutations were primarily scattered throughout the catalytic domain (Figure 6). A patient could simultaneously harbor multiple mutations in the *SGK1* gene. Of the 138 patients with *SGK1* mutations, 57 had one *SGK1* mutation, 48 had 2 to 5 *SGK1* mutations, and 33 had six or more *SGK1* mutations. The largest number of *SGK1* mutations identified in a patient was 23.

## 4 DISCUSSION

Next‐generation sequencing is widely used in clinical cancer research. The relationship between gene mutations and the development and prognosis of DLBCL is a research hotspot. More than 100 driver genes have been identified in DLBCL, and common mutations have been identified. However, key issues, such as how to interpret the mutation data and the clinical significance of the mutations, remain poorly understood and require urgent attention. Several recent studies have sought to introduce mutation data into the molecular classification of DLBCL to investigate the relationships between gene mutations and COO and prognosis. The use of gene mutations for molecular classification of DLBCL was first reported by Schmitz et al.^1^ However, 53.4% of the patients in the Schmitz et al. study could not be classified, indicating the limitations of their classification system. The Chapuy et al.^2^ dataset published in 2018 reported a comprehensive genetic analysis of 304 DLBCL cases. Based on gene mutation data, alterations in copy number, and structural variations, patients with DLBCL in this study were classified into five subsets (C1–C5). Specifically, C1 and C5 were predominantly of the ABC origin, C3 and C4 were predominantly of the GCB origin, C2 was of both ABC and GCB origins, and C0 lacked detectable genetic drivers. Analysis of the relationships between the new genotypes and patient prognosis indicated that patients in the C0, C1, and C4 subsets had more favorable prognosis compared to the patients in the C3 and C5 subsets who had poor prognosis. The study published by Lacy et al.^3^ in 2020 analyzed the gene mutation signatures of 928 patients with DLBCL by targeted sequencing of 293 genes, and divided the patients into five clusters: MYD88, BCL2, SOCS1/SGK1, TET2/SGK1, and NOTCH2. Among them, the MYD88 and BCL2 clusters were mainly of ABC origin, SOCS1/SGK1 and TET2/SGK1 clusters were mainly of GCB origin, and NOTCH2 comprised a mixture of ABC, GCB, and unclassified cases. The 5‐year OS rates of patients in the MYD88, NOTCH2, NEC, BCL2, SOCS1/SGK1, and TET2/SGK1 clusters were 42%, 48.1%, 53.6%, 64.9%, 62.5%, and 60.1%, respectively. Based on the 5‐year survival rates, there was no significant difference among BCL2, SOCS1/SGK1, and TET2/SGK1, suggesting that the classification had suboptimal prognostic utility. Both the Chapuy et al.^2^ and Lacy et al.^3^ datasets were retrospective studies that clustered patients with different genetic signatures using computational algorithms. The methods used by these studies were complicated, and standardized classification criteria were lacking. A patient often harbored multiple mutations that belonged to different subtypes. These facts rendered classification difficult and posed challenges to the widespread prospective application of the classification system in the clinic.

To gain further insight into the clinical significance of the DLBCL driver gene mutations, we reinterpreted the abovementioned studies. The Lacy et al. dataset^3^ comprised a large sample size, with detailed clinical and gene mutation data. We divided their cohort into high‐ and low‐risk groups based on the presence of POD24 and found that the mutation frequencies of 11 genes, including *MYD88*(L265P), *SGK1*, *MPEG1*, *TP53*, *SPEN*, *NOTCH*, *ETV6*, *TNFRSF14*, *MGA*, *CIITA*, and *PIM1*, were significantly different between the high‐ and low‐risk groups. Analysis of the relationship with patient survival showed that patients with *SGK1* mutations had the best prognosis. We then utilized data from the Reddy et al.^6^ and Chapuy et al.^2^ datasets and confirmed that patients with *SGK1* mutations had a good prognosis. The mutation frequency of *SGK1* in DLBCL is 10%–16%.^2^, ^3^, ^6^ Although *SGK1* mutation is considered as common, its clinical significance is poorly understood. Furthermore, the prognostic impact of *SGK1* mutations has not been reported. Therefore, we focused on delineating the significance of *SGK1* mutations in DLBCL. We found that most patients with *SGK1* mutations had DLBCL originating from GCB. Further analysis showed that among patients with GCB DLBCL, those with *SGK1* mutations exhibited a better prognosis than those without *SGK1* mutations. In the Lacy et al.^3^ dataset, most patients with *SGK1* mutations were classified into the SOCS1/SGK1 and TET2/SGK1 clusters. The COO in these two clusters were primarily GCB, and the patients in these two clusters also had a good prognosis. However, these two clusters included many patients without *SGK1* mutations. Thus, we regrouped the patients in both clusters, such that patients with *SGK1* mutations were in one group. We then compared the prognosis data for patients with and without *SGK1* mutations in the SOCS1/SGK1 and TET2/SGK1 clusters. We found that the patients in these two clusters without *SGK1* mutations had a poorer prognosis than those with *SGK1* mutations, suggesting that these two clusters could be further stratified by the *SGK1* mutation status. In the Chapuy et al.^2^ dataset, the C3 and C4 subsets were comprised predominantly of the GCB DLBCL subtype. We analyzed the C3 and C4 subsets in the study and found that among patients in the C3 and C4 subsets who were administered the R‐CHOP regimen, those with *SGK1* mutations exhibited a better prognosis than those without *SGK1* mutation. This suggests that the C3 and C4 subsets can be further stratified by the *SGK1* mutation status.

Most *SGK1* mutations were single‐base substitutions, which were scattered throughout the catalytic domain of the enzyme. Additionally, multiple *SGK1* mutations could be identified in any one patient. Notably, *SGK1* has been reported to exhibit oncogenic properties.^9^ Therefore, *SGK1* may play an oncogenic role in DLBCL development and progression with the mutation leading to its inactivation. Therefore, the *SGK1* mutation appears to be a promising molecular marker for prognosis in DLBCL and occurs predominantly in the GCB DLBCL subtype.

The origin of GCB DLBCL is the B cells in the germinal center (GC). The GC is a special microenvironment in secondary lymphoid tissues, where antigen‐activated B cells undergo clonal expansion, immunoglobulin class switching, and affinity maturation. GC cells repeatedly migrate between the dark zone and light zone of lymphoid follicles. These cells undergo clonal expansion and somatic hypermutation in the dark zone, followed by B‐cell receptor affinity selection in the light zone.^10^, ^11^, ^12^, ^13^

*SGK1* is a serine/threonine kinase in the AGC kinase family and shares high homology and many kinase functions with the Akt family.^14^ *SGK1* was originally cloned from rat mammary tumor cells stimulated by serum and glucocorticoids. Its function is closely associated with the phosphorylation of mammalian target of rapamycin (mTOR).^15^ *SGK1* transforms into an open conformation upon phosphorylation by mTOR at Ser422 and becomes fully activated by PDK1.^16^, ^17^ *SGK1* has been implicated in numerous physiological and pathological processes and plays an important role in oncology. *SGK1* is a crucial Akt‐independent regulator of the PI3K/mTOR signaling pathway which is involved in the regulation of cancer growth, survival, metastasis, autophagy, immunomodulation, cancer stem cells, cell cycle, and induction of therapeutic resistance. Very recently, Gao and colleagues reported that there were some mutations with enhanced function, the splice mutants, nonsense and frameshift variants within exon‐1 result in translation from downstream methionine that exclude the degradation domain and thereby generate stabilized SGK1 protein isoforms.^18^ The relationship between gain of function mutation and prognosis is unclear and needs further study.

Several studies on *SGK1* revealed that its expression is elevated in a multitude of cancers and was found to be associated with cancer growth, survival, and metastasis.^19^, ^20^, ^21^, ^22^ *SGK1* is essential for the proliferation of cancer cells that rely on PI3K activation, and *SGK1* deficiency reduces the proliferation and viability of cancer cells in various malignant cancers.^23^, ^24^, ^25^, ^26^, ^27^ Combined inhibition of *SGK1* and Akt has been shown to be more effective in suppressing cell growth than in inhibiting either PI3K or Akt alone.^26^ *SGK1* has been shown to induce resistance to chemo‐ and radiotherapy in many human cancers,^28^ whereas an *SGK1* inhibitor significantly increased the apoptosis of colon cancer and breast cancer cells following radiotherapy.^23^, ^29^

In DLBCL, mutation of *SGK1* may lead to its loss of function, rendering lymphoma cells more sensitive to glucocorticoids, chemotherapy drugs, and radiotherapy, thereby improving prognosis. Inhibition of *SGK1* activity may be a potential anticancer treatment approach, particularly for GCB DLBCL. Previous studies focused on identifying genetic markers associated with poor prognosis; however, the recent Flyer^30^ and S1001^31^ trials showed that de‐escalating therapy may be appropriate for low‐risk DLBCL. Therefore, identification of a low‐risk marker is important for eliminating short‐ and long‐term toxicities. *SGK1* mutation can define a group of patients with DLBCL with favorable prognosis. However, whether the intensity of chemotherapy can be reduced in this group of patients requires further analysis.

## CONFLICT OF INTEREST

The authors declare that they have no conflict of interest.

## AUTHOR CONTRIBUTIONS

B.P.G., Y.J.H., and Y.D. were responsible for data analysis, interpretation, mapping, and drafting of manuscripts. B.P.G., Y.J.H., and Y.D. contributed equally to this article. C.C.L. is responsible for screening and collating the data. H.C. supervised the whole analysis and provided guidance and instructions. All authors read and approved the final manuscript.

## ETHICS STATEMENT

As the data used in this study are publicly available, no ethical approval was required.

## Supporting information


Dataset S1

Dataset S2

Dataset S3

## Data Availability

The datasets presented in this study can be found in online repositories.
